# New Books

**Published:** 2007-07

A New Ecology: Systems Perspective

S. Jorgensen, B. Fath, S. Bastianoni, J. Marques, F. Muller, S. Nielsen, B. Patten, E. Tiezzi, R. Ulanowicz

Burlington, MA:Elsevier, 2007. 288 pp. ISBN: 0-444-53160-2, $150

Apollo’s Fire

Jay Inslee, Bracken Hendricks

Washington, DC:Island Press, 2007. 336 pp. ISBN: 1-59726-175-0, $25.95

Bioinformatics

Andrzej Polanski, Marek Kimmel

New York:Springer, 2007. 376 pp. ISBN: 3-540-24166-9, $69.95

Chernobyl—What Have We Learned? The Successes and Failures to Mitigate Water Contamination Over 20 Years

Yasuo Onishi, Oleg V. Voltsekhovich, Mark J. Zheleznyak, eds.

New York:Springer, 2007. 289 pp. ISBN: 1-4020-5348-1, $129

Computational Toxicology: Risk Assessment for Pharmaceutical and Environmental Chemicals

Sean Ekins, ed.

Hoboken, NJ:John Wiley & Sons, 2007. 816 pp. ISBN: 0-470-04962-4, $140

Energy for Sustainability: Technology, Planning, Policy

John Randolph, Gilbert Masters

Washington, DC:Island Press, 2007. 524 pp. ISBN: 1-59726-103-3, $75

Energy in Nature and Society: General Energetics of Complex Systems

Vaclav Smil

Cambridge, MA:MIT Press, 2007. 512 pp. ISBN: 0-262-69356-9, $32

Environment, Energy, and Resources Law: The Year in Review 2006

Section of Environment, Energy, and Resources

Chicago:ABA Publishing, 2007. 398 pp. ISBN: 1-59031-881-1, $59.95

Global Climate Change and U.S. Law

Michael B. Gerrard, ed.

Chicago:ABA Publishing, 2007. 784 pp. ISBN: 1-59031-816-4, $59.95

Ignition: What You Can Do to Fight Global Warming and Spark a Movement

Jonathan Isham, Sissel Waage

Washington, DC:Island Press, 2007. 296 pp. ISBN: 1-59726-156-4, $18.95

Male-mediated Developmental Toxicity

D. Anderson, M. Brinkworth, eds.

New York:Springer, 2007. 305 pp. ISBN: 0-85404-847-2, $179

Mass Spectral and GC Data of Drugs, Poisons, Pesticides, Pollutants and Their Metabolites, 3rd ed.

Hans H. Maurer, Karl Pfleger, Armin Weber

Hoboken, NJ:John Wiley & Sons, 2007. 1,452 pp. ISBN: 3-527-31538-3, $750

Microarray Data Analysis: Methods and Applications

Michael J. Korenberg

Totowa, NJ:Humana Press, 2007. 312 pp. ISBN: 1-58829-540-8, $99.50

Molecular Microbiology of Heavy Metals

Dietrich H. Nies, Simon Silver, eds.

New York:Springer, 2007. 460 pp. ISBN: 3-540-69770-1, $229

NGO Diplomacy: The Influence of Nongovernmental Organizations in International Environmental Negotiations

Michelle M. Betsill, Elisabeth Corell, eds.

Cambridge, MA:MIT Press, 2007. 256 pp. ISBN: 0-262-02626-0, $50

Reinventing Los Angeles: Nature and Community in the Global City

Robert Gottlieb

Cambridge, MA:MIT Press, 2007. 464 pp. ISBN: 0-262-07287-4, $62

The Law and Policy of Ecosystem Services

J.B. Ruhl, Steve, E. Kraft, Christopher L. Lant

Washington, DC:Island Press, 2007. 368 pp. ISBN: 1-55963-094-9, $70

The Unnatural History of the Sea

Callum Roberts

Washington, DC:Island Press, 2007. 456 pp. ISBN: 1-597-26-102-5, $28

Towards a Cleaner Planet

Jaime Klapp, Jorge L. Cervantes-Cota, José Federico Chávez Alcalá, eds.

New York:Springer, 2007. 420 pp. ISBN: 3-540-71344-9, $169

Toxic Tort Litigation

D. Alan Rudin

Chicago:ABA Publishing, 2007. 491 pp. ISBN: 1-59031-734-3, $149.95

